# Editorial: Advancing Molecular Oncology in Mexico

**DOI:** 10.3390/ijms26125456

**Published:** 2025-06-06

**Authors:** Jorge Melendez-Zajgla, Ignacio Camacho-Arroyo, Mahara A. Valverde Ramirez, Mauricio Rodriguez-Dorantes

**Affiliations:** 1 Laboratorio de Genómica Funcional del Cáncer, Instituto Nacional de Medicina Genómica, Mexico City 14610, Mexico; 2 Unidad de Investigación en Reproducción Humana, Instituto Nacional de Perinatología-Facultad de Química, Universidad Nacional Autónoma de México (UNAM), Ciudad de México 04510, Mexico; 3 Departamento de Medicina Genómica y Toxicología Ambiental, Instituto der Investigaciones Biomédicas, Universidad Nacional Autónoma de México (UNAM), Ciudad de México 04510, Mexico; mahara@iibiomedicas.unam.mx; 4 Laboratorio de Oncogenómica, Instituto Nacional de Medicina Genómica, Mexico City 14610, Mexico; mrodriguez@inmegen.gob.mx

Cancer remains a formidable public health challenge globally, and Mexico is no exception. As the third leading cause of death in the country, malignant neoplasms are projected to rise in both morbidity and mortality in the coming years. This pressing issue has galvanized numerous research groups across Mexico to delve into the molecular intricacies of various cancers, including breast, prostate, lung, colon, and cervical cancers, as well as glioblastomas, leukemia, and pancreatic cancer. The special issue of the *International Journal of Molecular Sciences* titled “State-of-the-Art Molecular Oncology in Mexico” host works showing the latest advancements and research efforts in this critical field.

## 1. The Landscape of Molecular Oncology in Mexico

As a middle-income country in a current demographic transition, Mexico has both low-income and high-income health problems, so an increase in non-transmisible diseases is currently happening. Among these diseases, cancer is probably the most studied. Nevertheless, the complexity of cancer in Mexico is heightened by unique population and environmental factors, which differ from other countries. Examples of this are the high prevalence of Cervical Cancer, the high incidence of Pediatric Leukemias and the differences in the mutational landscapes of Lung and other tumors. These differences difficult the extrapolation of overseas cancer research to the country. However, the advent of molecular biology and high-throughput technologies has significantly transformed traditional oncology studies and provide new avenues for clinical translational research. These innovations have enhanced cancer diagnosis, prognosis, and therapy, marking a new era in cancer research and treatment. Mexican researchers are at the forefront of these developments, contributing valuable insights that hold promise for translational medicine and diverse clinical applications.

The special issue features 17 published papers that provide a comprehensive overview of the current state of molecular oncology research in Mexico; six are reviews with basic science approaches, epidemiological analysis and promising treatments. From the original works, here are some notable contributions:

1. Pancreatic Cancer Cells Induce MicroRNA Deregulation in Platelets. Pancreatic cancer has a high mortality rate due to late detection, making early diagnostic biomarkers crucial. This study investigates the potential of liquid biopsies, specifically educated platelets, for early detection. Platelets interact bidirectionally with tumor cells, affecting cancer cell behaviors. The research showed that co-culturing platelets with pancreatic cancer cells increased cell proliferation, migration, clonogenicity, and stemness marker expression (Nanog, Sox2, Oct4). Additionally, differential microRNA (miRNA) expression was observed in platelets from pancreatic cancer patients compared to healthy subjects. Notably, differences in miRNA profiles were found between blood-derived and pancreatic juice-derived platelets in cancer patients, indicating distinct platelet subpopulations that merit further study [1].
2. The Transcriptomic Landscape of Pediatric Astrocytomas. Central nervous system tumors in children are common and astrocytomas are the most diagnosed type. New molecular classification based on genes like IDH1/2 and H3F3A is added to the traditional histological classification. RNA sequencing revealed shared misregulated genes in pediatric astrocytomas, suggesting potential implications in tumorigenesis [2].
3. c-Kit Induces Migration of Triple-Negative Breast Cancer Cells and Is a Promising Target for Tyrosine Kinase Inhibitor Treatment. The researchers conclude that c-Kit is a targetable molecule in triple-negative breast cancer (TNBC) and that Nilotinib may be a promising candidate drug for its treatment. This study provides new insights into potential targeted therapies for TNBC, which currently lacks specific treatment options beyond chemotherapy [3].
4. Characterization of Philadelphia-like Pre-B Acute Lymphoblastic Leukemia: Experiences in Mexican Pediatric Patients. This study proposes a diagnostic strategy for Philadelphia chromosome-like (Ph-like) acute lymphoblastic leukemia (ALL) in Mexican patients, addressing the challenges of limited genomic technology resources. The researchers analyzed 104 pre-B ALL patients without recurrent gene fusions using various methods, including gene expression profiling, CRLF2 analysis, and screening for specific gene rearrangements. They found that 38.5% of patients were classified as Ph-like, with a high frequency of CRLF2 abnormalities and Jak2-Stat5 associated abnormalities. The proposed diagnostic approach involves analyzing CRLF2 overexpression/genetic lesions, Abl phosphorylation of surrogate markers, gene rearrangements, and Sanger sequencing. This strategy provides a practical method for identifying Ph-like ALL in Mexican patients, potentially improving diagnosis and treatment options in regions with limited access to advanced genomic technologies [4].
5. Structural Basis of the Binding Mode of the Antineoplastic Compound Motixafortide (BL-8040) in the CXCR4 Chemokine Receptor. This study investigates the molecular mechanism of motixafortide, a promising CXCR4 antagonist, using computational techniques including molecular dynamics simulations. The researchers found that motixafortide favors inactive conformations of CXCR4, while the agonist CXCL12 triggers active-like conformations. Motixafortide’s six cationic residues form charge-charge interactions with acidic CXCR4 residues, and two synthetic bulky chemical moieties work together to restrict conformations of important residues associated with CXCR4 activation. These findings elucidate how motixafortide interacts with and stabilizes CXCR4’s inactive state, providing valuable insights for the rational design of CXCR4 inhibitors with similar pharmacological properties [5].
6. Allopregnanolone Promotes Migration and Invasion of Human Glioblastoma Cells through the Protein Tyrosine Kinase c-Src Activation This study investigated the effects of allopregnanolone (3α-THP), a progesterone metabolite, on glioblastoma (GB) cell migration and invasion. The researchers found that 3α-THP promoted migration and invasion in multiple human GB cell lines at a concentration of 100 nM. While GB cells showed higher levels of AKR1C1-4 enzymes compared to normal astrocytes, silencing these enzymes did not affect 3α-THP’s impact on cell migration and invasion Importantly, 3α-THP rapidly activated c-Src protein in GB cells, and pharmacological inhibition of c-Src reduced the pro-migratory and pro-invasive effects of 3α-THP. These findings suggest that 3α-THP promotes GB migration and invasion primarily through c-Src activation [6].

## 2. Conclusions

The “State-of-the-Art Molecular Oncology in Mexico” special issue represents a significant milestone in cancer research, highlighting the innovative work being conducted by Mexican scientists. By fostering collaboration and sharing cutting-edge findings, this special issue aims to drive forward the understanding and treatment of cancer, ultimately improving patient outcomes and public health in Mexico and beyond.
